# Comparison of Information Criteria for Detection of Useful Signals in Noisy Environments

**DOI:** 10.3390/s23042133

**Published:** 2023-02-14

**Authors:** Leonid Berlin, Andrey Galyaev, Pavel Lysenko

**Affiliations:** Institute of Control Sciences of RAS, 117997 Moscow, Russia

**Keywords:** information entropy, signal-to-noise ratio, statistical complexity

## Abstract

This paper considers the appearance of indications of useful acoustic signals in the signal/noise mixture. Various information characteristics (information entropy, Jensen–Shannon divergence, spectral information divergence and statistical complexity) are investigated in the context of solving this problem. Both time and frequency domains are studied for the calculation of information entropy. The effectiveness of statistical complexity is shown in comparison with other information metrics for different signal-to-noise ratios. Two different approaches for statistical complexity calculations are also compared. In addition, analytical formulas for complexity and disequilibrium are obtained using entropy variation in the case of signal spectral distribution. The connection between the statistical complexity criterion and the Neyman–Pearson approach for hypothesis testing is discussed. The effectiveness of the proposed approach is shown for different types of acoustic signals and noise models, including colored noises, and different signal-to-noise ratios, especially when the estimation of additional noise characteristics is impossible.

## 1. Introduction

Since Shannon [1] introduced information and information entropy, these concepts have attracted significant attention from scientists, as evidenced by the large number of articles devoted to the development of information theory in relation to various theoretical and practical aspects. Many different information criteria, metrics, and methods for their calculation that are based, one way or another, on the concepts of Shannon entropy have been proposed and investigated [2]. These metrics can be used quite successfully in signal processing, which eventually led to the emergence of a separate section of this scientific area, called entropic signal analysis [3].

For signals described by time series, the information entropy can be calculated on the basis of both signal representation in the time domain [4] and its representation in the frequency domain [5], i.e., using the signal spectrum. The convenience of the second approach comes from the fact that white noise, which is usually used to model background noise in these problem statements, has a uniform frequency distribution. This allows us to simplify its mathematical description and separate useful signals more effectively.

Decision theory considers change-point detection problems, which are closely related to the problems discussed above: often, in such problems, the moment of change in the parameters of a random process registered in discrete time must be determined. In [6,7], many probabilistic-statistical methods of solving such problems are considered. The Neyman–Pearson approach to this problem was used in [8]. Additionally, one cannot ignore the so-called Anomaly Detection Problems, where detection of anomalies in time series is required [9,10,11], i.e., the moment at which the behavior of the system begins to qualitatively differ from normal for various reasons, in particular, due to unwanted external interference. The electrocardiogram (ECG) is one example of such a time series, and ECGs have been analyzed in a large number of articles, for example, [12]. The presence of an anomaly in this case can indicate health problems, and detection at an early stage may save the life of the patient.

Of particular interest is the processing of acoustic signals, which can be useful, for example, in Voice Activity Detection (VAD) problems [13] related to voice assistants. The task is usually to separate speech segments from background environmental noise. Related articles [5,14,15] present a method for endpoint detection, i.e., the determination of the limits of a speech signal in a mixture of this signal and background noise based on the calculation of the spectral entropy. The general idea of methods based on information criteria is that their values experience a sharp jump when a useful signal appears in the noise.

In a series of articles [16,17,18,19,20,21], researchers introduced the concept of a statistical measure of signal complexity, which they called statistical complexity. In [22,23], statistical complexity and information entropy were used to classify various underwater objects of animate and inanimate nature from recorded sound. In the present article, we use this measure to indicate the appearance of an useful acoustic signal in a highly noisy mixture. It should be noted that the positive side of the proposed method is that it does not require any a priori knowledge about the signal to be detected. However, a priori information, such as the approximate frequency range of the signal is known, its detection will be even more accurate.

The structure of the paper is as follows. Section 2 provides a brief theoretical summary of the information criteria used in various known signal detection methods. In Section 3, entropy variation is investigated and statistical complexity is introduced. In Section 4, the connection between statistical complexity and the Neyman–Pearson criterion for hypothesis testing is also discussed to justify the proposed approach. Section 5 is provides a variety of examples, gives a comparison of different information criteria, and discussed the results, which allows us to make an educated choice about a suitable rule for the detection of signals in a noisy mixture. Section 6 summarizes the conducted research and provides the direction for future work.

## 2. Information Criteria

### 2.1. Information Entropy and Other Information Criteria

In information theory, the entropy of a random variable is the average level of ”surprise” or ”uncertainty” inherent in the possible outcomes of the variable. For a discrete random variable *X* that takes values in the alphabet X and has a distribution density of *p*p:X→[0,1], the entropy, according to Shannon [1], is defined as

(1) $$H\left(p\right)=-\sum_{x\in X}p\left(x\right)\log_{2}p\left(x\right),$$

where ∑ denotes the sum across all possible values of the variable. When computing the sum (1), it is agreed that 0log0=0, and this assumption holds for all future equations. From the Formula (1), it follows that entropy reaches its maximum value when all states of the system are equally probable.

There are several definitions of information divergences, i.e., the statistical distances between two distributions. The Kullback–Leibler divergence (or mutual entropy) between two discrete probability distributions p(x) and q(x) on an event set X is defined as

(2) $$D_{KL}\left(pq\right)=\sum_{x\in X}p\left(x\right)\log_{2}\frac{p(x)}{q(x)}.$$

This measure is a statistical distance and distinguishes statistical processes by indicating how much p(x) differs from q(x) by the maximum likelihood hypothesis test when the actual data obey the distribution p(x). It is easy to see that

(3) $$D_{KL}\left(pq\right)=H\left(p,q\right)-H\left(p\right),$$

where H(p,q) is a cross-entropy between *p* and *q*:

(4) $$H\left(p,q\right)=E_{p}\left[-\log_{2}q\right],$$

where $E_{p}\left[\cdot \right]$ is an operator of the mathematical expectation relative to the distribution *p*.

The symmetrized Kullback–Leibler distance [13] is often used in studies:

(5) $$\rho \left(pq\right)=D_{KL}\left(pq\right)+D_{KL}\left(qp\right).$$

However, the Jensen–Shannon divergence, which symmetrizes the Kullback–Leibler divergence and is often a more convenient information measure for practical applications, is used more often:

(6) $$JSD\left(pq\right)=\frac{D_{KL}\left(pm\right)+D_{KL}\left(qm\right)}{2},\;m=\frac{p+q}{2}.$$

It is symmetric and always has a finite value. The square root of the Jensen–Shannon divergence is a metric that is often called the Jensen–Shannon distance.

It is easy to see that

(7) $$JSD\left(pq\right)=H\left(m\right)-\frac{1}{2}(H\left(p\right)+H\left(q\right)).$$

Another quantity related to the complexity of the system is the ”disequilibrium,” denoted by *D*, which shows the deviation of a given probability distribution from a uniform one. The concept of the statistical complexity of a system can be considered a development of the concept of entropy. In [16,17,18,19,20,21], it is defined as

(8) C=H·D,

where *C* is the statistical complexity, *H* is the information entropy, and *D* is a measure of the disequilibrium of the distribution relative to the uniform one.

The measure of statistical complexity reflects the relationship between the amount of information and its disequilibrium in the system. As a parameter *D*, according to the authors of [16], one can choose any metric that determines the difference between the maximum entropy and the entropy of the studied signal. The simplest example of disequilibrium is the square of the Euclidean distance in $R^{N}$ between the original distribution and the uniform distribution, but often, the Jensen–Shannon divergence [22,23] is also used.

### 2.2. Time Entropy

Now let us consider the information characteristics mentioned above in relation to time series. The Shannon entropy for systems with unequal probability states is defined as follows: Let the *i*-th state of the system have a probability of $p_{i}=N_{i}/N$, where *N* is a sample volume and $N_{i}$ is the amount of filling at the *i*-level. Then, the entropy H(p), according to the Formula (1), equals

(9) $$H\left(p\right)=-\sum_{i=1}^{N}p_{i}\log_{2}p_{i}.$$

From here, we consider discrete probability distributions $p_{i}$ with the following properties:

(10) $$p_{i}\in \left[0,1\right],\;\sum_{i=1}^{N}p_{i}=1.$$

There are different ways to calculate probabilities $p_{i}$ from the time series. The simplest one is as follows: First, the maximum $x_{\max}$ and minimum $x_{\min}$ values are found for the considered time series x(t) with *N* data points. Then, the interval ($x_{\max}-x_{\min}$) is divided into *n* subintervals (levels) so that the value of the interval Δx is not less than the confidence interval of the observations. The resulting sample is treated as a “message”, and the *i* subintervals are treated as an “alphabet”. Then, we find the number $\Delta N_{i}$ of sample values $x_{k}$ that fall into each of the subintervals and determine the relative population level $p_{i}^{t}$ (the probability of a value from the sample falling into a subinterval *i*, that is, the relative frequency of occurrence of the “letter” in the “message”):

(11) $$p_{i}^{t}=\frac{\Delta N_{i}}{N},\;\sum_{i=1}^{n}\Delta N_{i}=N,\;\sum_{i=1}^{n}p_{i}^{t}=1.$$

The elementary entropy of the sampling is defined as the Shannon entropy (9) on a given set $p_{i}^{t}$, and this is normalized to the total number of states *n* so that its values belong to the interval [0,1]:

(12) $$H\left(p^{t}\right)=\frac{-\sum_{i=1}^{n}p_{i}^{t}\log_{2}p_{i}^{t}}{\log_{2}n}.$$

This approach is known as the first sampling entropy [4] and is used, for example, in [24] to detect the hydroacoustic signals emitted by an underwater source.

On the other hand, the second sampling entropy can be defined as

(13) $$H\left(p^{0}\right)=\frac{-\sum_{i=1}^{N}p_{i}^{0}\log_{2}p_{i}^{0}}{\log_{2}N},\;p_{i}^{0}=\frac{x(t_{i})}{\sum_{k=1}^{N}x\left(t_{k}\right)}.$$

In this case, the signal samples themselves are considered “letters”, which are distributed across the time axis in contrast to the amplitude axis from (12), and the “alphabet” is the whole set of amplitudes.

### 2.3. Spectral Entropy

In addition to the time domain, the entropy can be calculated based on the representation of the signal in the frequency domain, i.e., $p_{i}$ can be calculated with the spectrum of the signal. Spectral entropy is a quantitative assessment of the spectral complexity of the signal in the frequency domain from an energy point of view.

Consider the time series x(t) and its spectral decomposition in the frequency domain $X(f_{i})$ with $N_{fft}$ frequency components, obtained using the Fast Fourier Transform (FFT). The spectral power density is estimated as follows:

(14) $$s\left(f_{i}\right)=\frac{1}{N_{fft}}X{\left(f_{i}\right)}^{2}.$$

Then, the probability distribution of the spectral power density $p^{s}=\left\{p_{1},p_{2},\ldots ,p_{N_{fft}}\right\}$ can be written in the form

(15) $$p_{i}^{s}=\frac{s(f_{i})}{\sum_{k=1}^{N_{fft}}s\left(f_{k}\right)},\;i=1,\ldots ,\;N_{fft},$$

where $s(f_{i})$ is the spectral energy for the spectral component with a frequency of $f_{i}$, $p_{i}^{s}$ is the corresponding probability density, $N_{fft}$ is the number of spectral components in the FFT, and the upper index *s* shows that the distribution refers to the signal spectrum. The resulting function is a spectrum distribution density function.

Finally, the spectral entropy can be determined with the Equation (9) and normalized by the size of the spectrum:

(16) $$H\left(p^{s}\right)=\frac{-\sum_{k=1}^{N_{fft}}p_{k}^{s}\log_{2}p_{k}^{s}}{\log_{2}N_{fft}}.$$

The Spectral Information Divergence (SID) method [25] was recently added to the Matlab mathematical package, and is calculated according to Formula (5) according to the similarity of two signals based on the divergence between the probability distributions of their spectra:

(17) $$SID\left(r,t\right)=\sum_{i}p_{i}\log\frac{p_{i}}{q_{i}}+\sum_{i}q_{i}\log\frac{q_{i}}{p_{i}},$$

where *r* and *t* are the reference and test spectra, respectively, and the values of the probability distribution $p_{i}$ and $q_{i}$ for these spectra are determined according to (15).

## 3. Entropy Variation and Related Information Criteria

The purpose of this section is to determine the most appropriate formulas for calculating the information criteria that are responsible for the differences between distributions. Let us consider an entropy variation with respect to the variation in the probability distribution. The following lemma is valid:

**Lemma** **1.** *For small variations $\delta q_{i}=p_{i}-q_{i}$ in the discrete distribution $q_{i}$, such as $p_{i}$, there is also some discrete distribution (10), and the decomposition of the entropy variation δH in the case of series convergence by powers $\delta q_{i}$ has the form*

(18) $$\delta H=H\left(q+\delta q\right)-H\left(q\right)=LH\left(pq\right)-\frac{D(p,q)N}{2\ln2}+o\left({\left(\delta q\right)}^{2}\right),$$

*The first summand of the entropy variation decomposition δH is the difference between cross-entropy and entropy, and the second depends on the weighted squares of the variation of the distribution:*

(19) LH(pq)=H(p,q)−H(q),

(20) $$D\left(p,q\right)=\frac{1}{N}\sum_{i=1}^{N}\frac{{\left(\delta q_{i}\right)}^{2}}{q_{i}}=\frac{1}{N}\sum_{i=1}^{N}\frac{{\left(p_{i}-q_{i}\right)}^{2}}{q_{i}}=\frac{1}{N}\left(\sum_{i=1}^{N}q_{i}{\left(\frac{p_{i}}{q_{i}}-1\right)}^{2}\right).$$

The proof of the Lemma 1 is given in Appendix A.

**Remark** **1.** *If q is the uniform distribution, i.e., $q_{i}=1/N$ for i=1,...,N, when*

LH(pq)=H(p,q)−H(q)=0,

*and the disequilibrium D=D(p,q) is proportional to variance of the distribution p relative to the uniform one and is equal to*

(21) $$D_{SQ}\left(p,q\right)=\sum_{i=1}^{N}{\left(p_{i}-\frac{1}{N}\right)}^{2}=\sum_{i=1}^{N}p_{i}^{2}-\frac{1}{N}.$$

Equation (21) coincides with the disequilibrium definition from [16].

According to Lemma 1 and Remark 1, we can introduce a new definition.

**Definition** **1.** *In the case where q is the uniform distribution statistical complexity, as defined in [16], it is proportional to the first nonzero member of the row of square entropy variation, namely,*

(22) $$C_{SQ}=H\left(p\right)\cdot D_{SQ}\left(p,q\right)=\left(-\sum_{i=1}^{N}p_{i}\log_{2}p_{i}\right)\cdot \left(\sum_{i=1}^{N}{\left(p_{i}-\frac{1}{N}\right)}^{2}\right).$$

**Remark** **2.** *In general cases, the statistical complexity is defined as*

(23) $$C∼\delta {\left(H\left(q\right)\right)}^{2}∼H\left(q\right)\delta H\left(q\right)=H\left(H_{max}-H\right),$$

*where $H_{max}$ is an entropy maximum.*

It follows from Remark 1 that the disequilibrium (21) and the complexity (22) concelts must be applied when evaluating and comparing signals with background noise that has a spectral distribution close to uniform.

The formula for disequilibrium (21) proposed in [16] is derived from entropy variation, but most papers use the Jensen–Shannon divergence [22] for disequilibrium:

(24) $$D_{JSD}=JSD\left(pq\right),$$

where $q_{i}=1/N$. The statistical complexity, correspondingly, is expressed as

(25) $$C_{JSD}\left(p\right)=H\left(p\right)\cdot JSD\left(pq\right).$$

Further on in the article, a comparison of the complexity graphs calculated for these two values $D_{SQ}$ and $D_{JSD}$ is presented.

Considering the signal distribution in the frequency and time domains, it can be observed that the spectral distribution does not require any additional estimation of the signal variance, whereas when calculating entropy in the time domain, variance estimation is required, since for white noise (Gauss distribution), the following formula is valid:

(26) $$H_{w}=-\int_{-\infty }^{+\infty }\rho^{t}\left(x\right)\log_{2}\left(\rho^{t}\left(x\right)\right)dx=\log_{2}\left(\sqrt{2\pi e}\sigma \right),$$

where $\rho^{t}\left(x\right)=\frac{1}{\sqrt{2\pi }\sigma }\exp\left(-{\left(x-\mu \right)}^{2}/\left(2\sigma^{2}\right)\right)$ is the Gaussian distribution.

**Remark** **3.** *For the case of two continuous distributions (p,q), the disequilibrium D(p,q) is equivalent to the f-divergence [26] with the quadratic function f:*

(27) $$D\left(p,q\right)=\int \rho_{q}\left(x\right){\left(\frac{\rho_{p}\left(x\right)}{\rho_{q}\left(x\right)}\right)}^{2}dx,$$

*if an integral exists.*

**Remark** **4.** *In the case of two Gaussian distributions with the parameters $\left(\mu_{p},\sigma_{p}\right)$ and $\left(\mu_{q},\sigma_{q}\right)$, Formulas (19) and (20) from Lemma 1 take the form*

(28) $$D\left(p,q\right)=\frac{\sigma_{q}^{2}}{\sigma_{p}\sqrt{2\sigma_{q}^{2}-\sigma_{p}^{2}}}\exp\left(\frac{{\left(\mu_{p}-\mu_{q}\right)}^{2}}{2\sigma_{q}^{2}-\sigma_{p}^{2}}\right)-1,$$

(29) $$LH\left(pq\right)=H\left(p,q\right)-H\left(q\right)=\frac{{\left(\mu_{p}-\mu_{q}\right)}^{2}}{2\sigma_{q}^{2}}+\frac{1}{2}\left(\frac{\sigma_{p}^{2}}{\sigma_{q}^{2}}-1\right).$$

The Formulas (28) and (29) are obtained by calculating the integrals (27) and (26) for continuous distributions. These are also applicable for discrete ones.

The problem addressed here is the determination of the most informative methods for calculating entropy and other information criteria. In our opinion, the answer can only be obtained in the presence of additional knowledge about the phenomenon under study. Indeed, when measuring the amplitude of the signal x(t), initially there is only knowledge of the time series samples $x(t_{i})$, i.e., we know the values of amplitudes in the increasing sequence of time samples $t_{i}$, i=1,…,N. Setting the distribution density using the Formula (11) itself allows some random variable to be defined.

Let us calculate the entropy $H(p^{0})$ by applying grouping (11) and considering the fact that the entropy does not change as the summation order changes. Thus, the following chain of equations is valid:

(30) $$\begin{array}{c} H\left(p^{0}\right)=-\sum_{i=1}^{N}p_{i}^{0}\log_{2}p_{i}^{0}\approx -\sum_{j=1}^{n}p_{j}^{1}\log_{2}\frac{p_{j}^{1}}{\Delta N_{j}}=\log_{2}N+H\left(p^{1}\right)-H\left(p^{1},p^{t}\right)\\ =\log_{2}N-D_{KL}\left(p^{1}p^{t}\right), \end{array}$$

where

(31) $$p_{j}^{1}=\sum_{i\in I_{j}}p_{i}^{0},\;\;I_{j}=\left\{i\in \left(1,\ldots ,N\right):p_{i}^{0}\in \left[\frac{j-1}{n}\max_{k}p_{k}^{0},\frac{j}{n}\max_{k}p_{k}^{0}\right]\right\},$$

so that

(32) $$p_{j}^{t}=\frac{\Delta N_{j}}{N},\;\;\Delta N_{j}=\sum_{i\in I_{j}}1.$$

Thus, we have obtained that

(33) $$H\left(p^{0}\right)\approx \log_{2}N-D_{KL}\left(p^{1}p^{t}\right).$$

If $p_{j}^{t}=\frac{\Delta N_{j}}{N}=\frac{1}{n}$ is now a uniform distribution, then (33) takes the form

(34) $$H\left(p^{0}\right)=\log_{2}N-\log_{2}n+H\left(p^{1}\right).$$

The Formula (33) shows the relationship between the entropy calculated from the time samples and the Kullback–Leibler distance between the distributions obtained by alphabetical grouping along the amplitude and time coordinate axes. Therefore, the next statement is valid.

**Corollary** **1.** *The value of $D_{KL}\left(p^{1}p^{t}\right)$ is approximately a constant value, independent of the method of grouping when the number of letters of the alphabet is large enough when their number is in some interval of values.*

The following observation considering distribution $p^{1}$ is true.

**Remark** **5.** *If the sequence of samples $x(t_{i})$ has the property of ergodicity and the signal is represented by white noise, then if the number of letters of the alphabet from (31) is large enough, the density $p^{1}$ will be close to Gaussian.*

Remark 5 allows us to estimate $H(p^{0})$ using Equation (26) for the Gaussian $p^{1}$.

Now, there are four distribution densities at our disposal: three of them, $p^{0}$, $p^{1}$, and $p^{t}$, are related to the time domain and are determined by the Formulas (13), (31), and (32), and one, $p^{s}$, is related to the signal spectrum, calculated by the Formula (15).

With the presence of four distribution densities, the following criteria are considered simultaneously: the normalized information entropy *H*, defined by Formulas (12) and (16); the statistical complexity *C*, computed by (22) with a disequilibrium *D* (21); the Jenson–Shannon divergence JSD (6); the spectral information divergence SID (17); and the cross-entropy and entropy difference LH (19).

Since the spectral density is used to compare the signal/noise mixture with white noise, i.e., with a uniform distribution, all of the proposed criteria are applicable for this density. In the case of temporal distributions, the normalized information entropy *H*, which depends only on the distribution under study, and the difference of cross-entropy and entropy LH, calculated explicitly by the Formula (29) for $\mu_{p}=\mu_{q}$, are estimated.

On the basis of the numerical experiments performed, a conclusion is made about the quality of the criteria used and the limits of their applicability in the presence of the noise component of the signal.

## 4. Hypothesis Testing

The classical probabilistic approach to the study of the considered problem of the detection of useful signals against background noise is called binary hypothesis testing. The binary problem associated with the decision to receive only noise (hypothesis $\Gamma_{0}$) or to receive a mixture of a useful signal and noise (hypothesis $\Gamma_{1}$) is solved [8].

In the statistical decision theory [6], it is shown that, in signal detection in the presence of noise, the optimal decisive rule is based on a comparison of the likelihood ratio with some threshold. The Neyman–Pearson criterion is used to select the threshold in the absence of a priori probabilities of the presence and absence of a useful signal. The efficiency of the detection procedure using the Neyman–Pearson criterion is characterized by the probability of correct detection with a fixed probability of false alarms.

The solution to the problem of distinguishing between two hypotheses can be derived from the following variant of the Neyman–Pearson lemma.

**Lemma** **2** (Neyman-Pearson)**.** *Let there be a measurable function, called a decisive rule,*

(35) $$d\left(x_{1},\ldots ,x_{N}\right)=\left\{\begin{array}{c} 1,\;the\;hypothesis\;\;\Gamma_{0},\\ 0,\;the\;hypothesis\;\;\Gamma_{1}, \end{array}\right.$$

*based on which*

$$\alpha \left(d\right)=Probability\;(of\;detection\;\Gamma_{0}\;\Gamma_{1}\;is\;true),$$

$$\beta \left(d\right)=Probability\;(of\;detection\;\Gamma_{1}\;\Gamma_{0}\;is\;true).$$

The decisive rule $d^{*}$ is optimal if

(36) $$\alpha \left(d^{*}\right)+\beta \left(d^{*}\right)=\underset{d}{\inf}\left[\alpha \left(d\right)+\beta \left(d\right)\right]=Er\left(N;\Gamma_{0},\Gamma_{1}\right),$$

*where $Er(N;\Gamma_{0},\Gamma_{1})$ is called an error function.*

In the problem of the detection of a useful signal, α is known as the probability of a false alarm occurring, and β is known as the probability of missing a useful signal.

The error function can be calculated precisely through the variation of the measure (with a sign) by the following formula from [6]:

(37) $$Er\left(N;\Gamma_{0},\Gamma_{1}\right)=1-\frac{1}{2}∥P_{0}^{\left(N\right)}-P_{1}^{\left(N\right)}∥=1-TV\left(P_{0},P_{1}\right),$$

where $P_{0}^{\left(N\right)}$ is the multivariate distribution function of observational statistics under hypothesis $\Gamma_{0}$, and $P_{1}^{\left(N\right)}$ is the multivariate distribution function of observational statistics under hypothesis $\Gamma_{1}$, and TV is the full variation $TV\left(P_{0},P_{1}\right)=\frac{1}{2}∥P_{0}^{\left(N\right)}-P_{1}^{\left(N\right)}∥$.

The peculiarity of the Formula (37) is that if the carriers on which the hypothesis measures $\Gamma_{0}$ and $\Gamma_{1}$ are concentrated are different, then $Er(N;\Gamma_{0},\Gamma_{1})\approx 0$. If the measures $P_{0}^{\left(N\right)}$ and $P_{1}^{\left(N\right)}$ are similar, then $∥P_{0}^{\left(N\right)}-P_{1}^{\left(N\right)}∥\approx 0$, and then $Er(N;\Gamma_{0},\Gamma_{1})\approx 1$.

For the problem of detecting a deterministic useful signal, the case $∥P_{0}^{\left(N\right)}-P_{1}^{\left(N\right)}∥=2TV\left(P_{0},P_{1}\right)\approx 0$ and the possibility of a reasonable estimate of this value is interesting.

The estimated TV(P,Q) constraints are known from thed estimate $\sqrt{JSD(PQ)}$, which is used to compute the statistical complexity $C_{JSD}$. Both TV and $\sqrt{JSD}$ are metrics related to the probability distribution space, but in the Euclidean space, $\sqrt{D_{SQ}}$ (21) serves as this metric. Since the problem of detecting a deterministic signal in the presence of background noise is considered, it is reasonable to additionally take into account this ”determinism” by multiplying $D_{SQ}$ by the entropy *H*, which is associated with the introduction and use of statistical complexity in the form of (22) and (25).

## 5. Modelling and Discussion

### 5.1. The Calculation Algorithm and Presentation of the Simulation Results

In all experiments, graphs of the information characteristics are presented as functions of time. The characteristics are calculated from the signal according to the following algorithm:

1. After being digitized with the sampling rate, the F audio signal is divided into short segments containing *W* digital samples.
2. The discrete densities $p_{i}$ (15) are calculated from the time or frequency domains.
3. The information criterion is calculated using $p_{i}$.
4. The sequence of values is displayed together with the signal on the time axis (each of the obtained values is extended by *W* counts).

When a certain threshold of the information criterion is exceeded, this indicates the appearance of a useful signal in the mixture.

The signal processing results according to this algorithm are presented below. For different acoustic signals, a comparison of the quality of indication of the appearance of a useful signal by different information criteria at different levels of added white noise is demonstrated. In addition, Section 5.5 shows a comparison of two methods for calculating the statistical complexity and draws conclusions about the usefulness of both.

The first acoustic signal chosen was an audio recording of a humpback whale song recorded underwater. A large set of such recordings is available from the Watkins Marine Mammal Sound Database collected by Woods Hole Oceanographic Institution and the New Bedford Whaling Museum. The ability to separate such signals from strong sea noise may be useful for research biologists for further classification and study. In addition, these signals are similar in structure to the human voice with separate words, the extraction of which could be useful, for example, in tasks of voice activity detection and speech recognition.

In all of the graphs presented below, the signal is marked with a blue line, and the corresponding information metric is marked with a red line. The left vertical axis corresponds to the values of the signal amplitude, and the right vertical axis corresponds to the values of the information metric. All horizontal axes represent the timeline in seconds. The signal is shown without added noise for better comprehension, but the variable parameter of the standard deviation $\sigma_{N}$ of the white noise is marked with a dashed line. All information metrics are normalized for the convenience of presentation. All calculations and visualizations were performed using Python. White, brown, and pink noises, which were artificially added to audio recordings, were also generated numerically.

### 5.2. Time Information Criteria

First, we consider the behavior of the information entropies $H(p^{t})$ and $H(p^{0})$, calculated from the time samples of the signal x(t).

**Figure 1:**
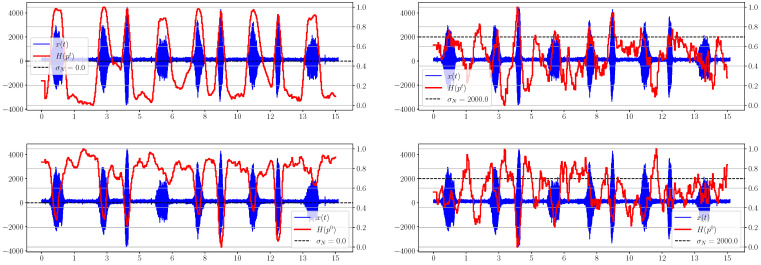
Graphs of $H(p^{t})$ and $H(p^{0})$ for different levels of added noise.

Figure 1 shows that as noise increases, there is serious degradation of the time entropy graph for both calculation methods, so that for $\sigma_{N}=2000$ (SNR ≈ 1.5 dB), these information criteria can no longer serve as reliable indicators of the appearance of a useful signal in the mixture. We note an interesting feature of the behavior of $H(p^{0})$ and $H(p^{t})$: the value of the first characteristic is maximal for the uniform distribution and decreases with the appearance of a useful signal in the mixture, while, in contrast, the value of the second is minimal in the absence of a signal and increases with its appearance. This obviously follows on from the formulas for calculating the distributions and entropies (11), (13), (12).

The information characteristic LH(pq) stands out favorably from the time entropies, as demonstrated above.

**Figure 2:**
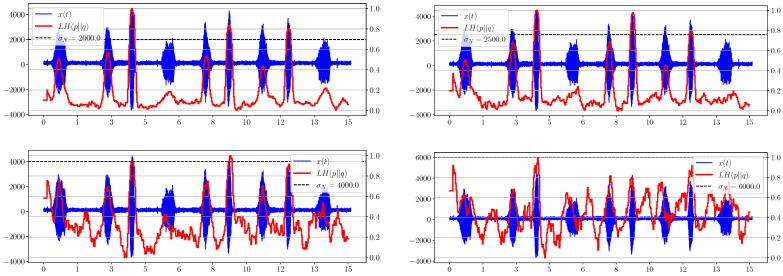
Graphs of LH(pq) for different levels of added noise.

In Figure 2, the LH for the noise level $\sigma_{N}=2000$ shows the appearance of a useful signal and works sufficiently, even for the double noise value. However, it should be noted that this is true only for stationary noise, whose average value does not change over time. Otherwise, this metric will react to changes in noise as well, which follows on from the formula (29). Moreover, initial estimation of $\sigma_{N}$ is required for the correct functioning of this criterion.

### 5.3. Time Entropy $H(p^{1})$

**Figure 3:**
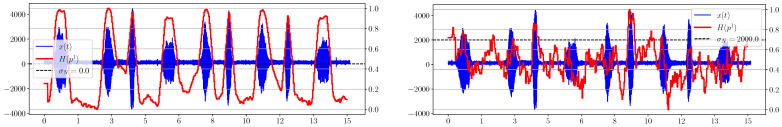
The entropy $H(p^{1})$ for the number of letters of the alphabet equal to 64.

**Figure 4:**
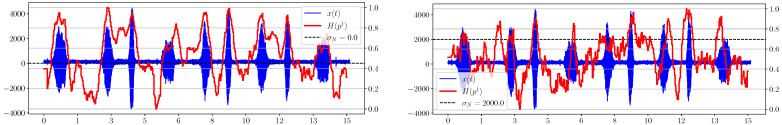
The entropy $H(p^{1})$ for the number of letters of the alphabet equal to 8.

The time entropy $H(p^{1})$ associated with another grouping of the ”alphabet” derived from the signal samples is considered separately and the graphs for different number of letters are shown in Figure 3 and Figure 4.

Changing the alphabet partitioning negatively affects the effectiveness of entropy in this representation:

### 5.4. Spectral Information Criteria

Information criteria based on the spectral distribution of $p^{s}$ are deprived of the disadvantages of the time criteria.

**Figure 5:**
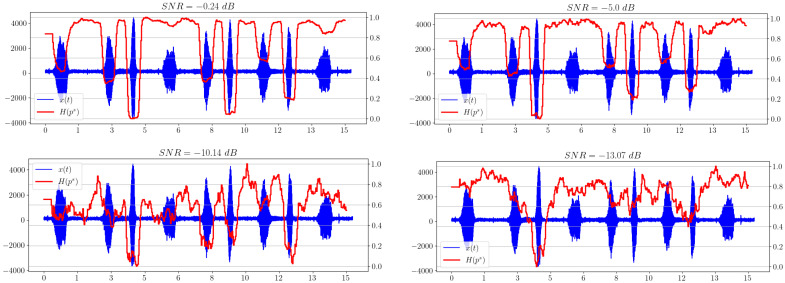
Spectral entropy plots for different SNRs.

Figure 5 shows the dependence of the spectral entropy on time. We can see a significant improvement in the maximum allowable noise level, at which the indication of the appearance of a useful signal is still possible, with respect to the graphs presented in Figure 2.

The point that we want to make is that the white noise in a signal in spectral representation has quite a definite uniform probability distribution, which greatly facilitates the calculation of entropy and saves us from the necessity of estimating the variance of this noise. Moreover, even if the noise is not stationary, i.e., its parameters change over time, in a small window *W*, it can still be considered white, and the above statement is still true.

**Figure 6:**
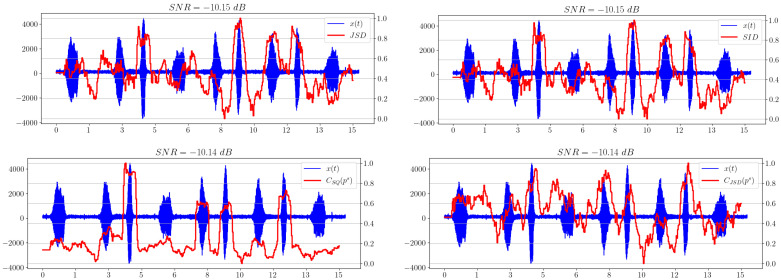
Information divergence plots based on spectral distribution.

The distribution $p^{s}$ can be used as the basis for a number of information divergences (17), (24), (22), (25):

Figure 6 shows that the separability of information metrics decreases along with the signal-to-noise ratio (SNR). However, the statistical complexity $C_{SQ}$ performs better than all other criteria, because it still allows a useful signal to be distinguished when other metrics behave irregularly and no longer show significantly excess levels compared to areas without a signal. Thus, it is the most promising characteristic in our opinion.

### 5.5. Comparison of Different Ways of Calculating the Statistical Complexity

**Figure 7:**
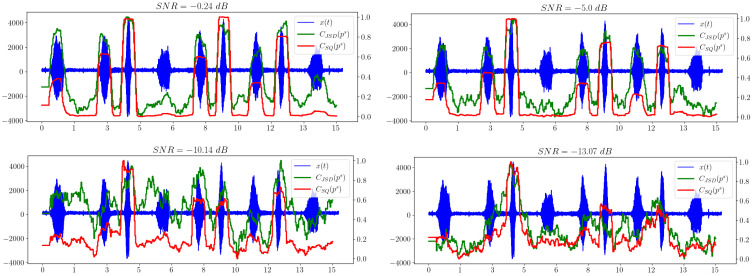
Comparison of statistical complexities $C_{SQ}$ and $C_{JSD}$.

Of separate interest is the comparison of the behavior of the statistical complexities $C_{SQ}$ and $C_{JSD}$, which essentially correspond to different methods of calculating the same value of statistical complexity. Figure 7 illustrates this comparison.

We can see that $C_{SQ}$ shows a better result when used as an indicator of the appearance of a useful signal in white noise compared to the Jensen–Shannon divergence.

### 5.6. Hydroacoustic Signal Model of an Underwater Marine Object

**Figure 8:**
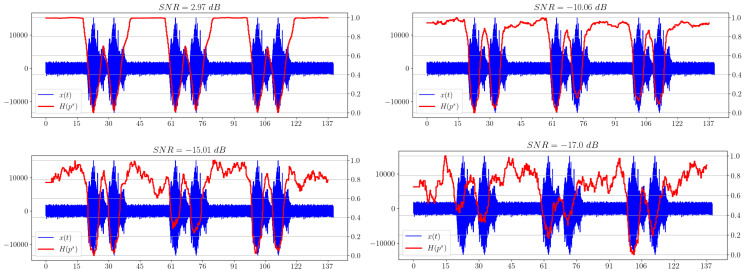
Spectral entropy plots for different SNRs.

The second signal is a modelled hydroacoustic signal of an underwater marine object. The study of such signals is important in military and civilian applications, because it can automate the process of analyzing the hydroacoustic scene and identifying potential threats. In Figure 8, spectral entropy dependencies for different levels of added noise are shown.

**Figure 9:**
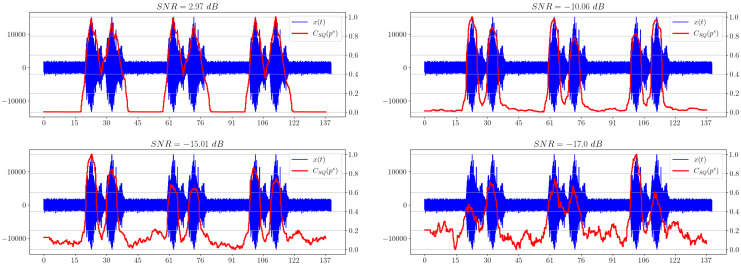
Graphs of statistical complexity for different SNRs.

Figure 9 shows the dependencies of statistical complexity for a given signal. It is worth noting that the selected information metric shows the presence of a useful signal, even for a very small SNR (≈−17 dB) in the last example.

It can be observed that, in comparison with all other information metrics, the statistical complexity shows the best result in terms of indicating the presence of a useful signal in the mixture, because it remains effective for small SNRs, while all other characteristics can no longer detect a useful signal in noisy receiving channels.

### 5.7. Hydroacoustic Signal Model with Pink Noise

**Figure 10:**
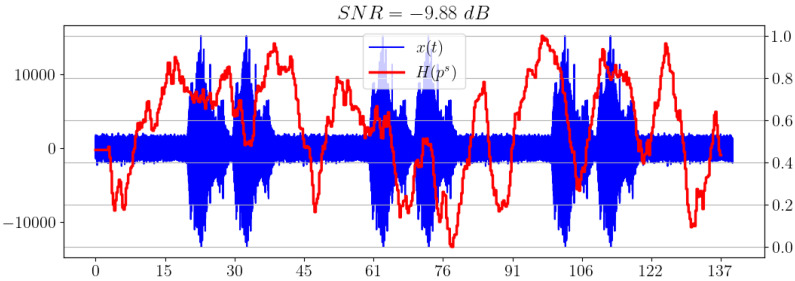
Spectral entropy for pink noise model.

Now let us change the additive noise model and use pink noise instead of white noise. As can be observed in Figure 10, the spectral entropy shows an unsatisfactory result for the chosen low SNR.

**Figure 11:**
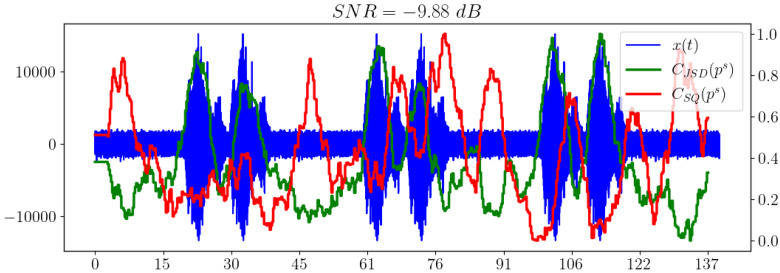
Statistical complexities $C_{SQ}$ and $C_{JSD}$ for pink noise model.

Figure 11 shows that, along with the spectral entropy, the statistical complexity $C_{SQ}$ performs poorly, but $C_{JSD}$ confidently shows the presence of a signal.

### 5.8. Hydroacoustic Signal Model with Brown Noise

**Figure 12:**
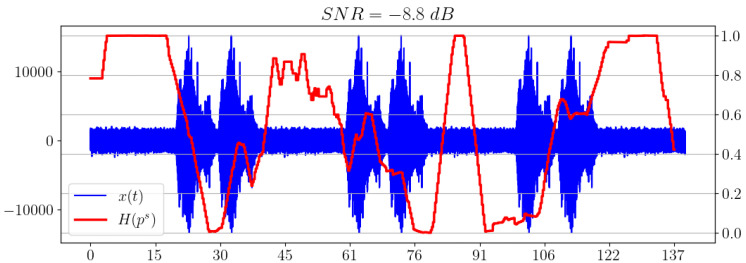
Spectral entropy for brown noise model.

In this example, brown noise is used as the noise model. As in the previous subsection, spectral entropy fails in the task of signal extraction, as shown in Figure 12.

**Figure 13:**
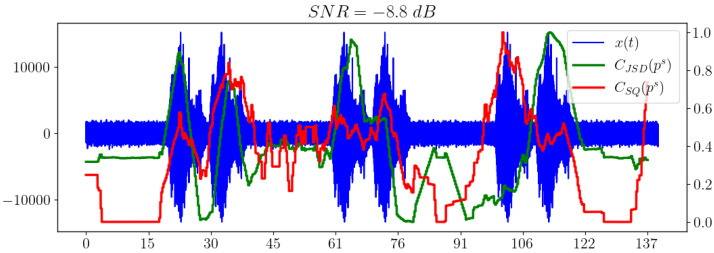
Statistical complexities $C_{SQ}$ and $C_{JSD}$ for brown noise model.

However, Figure 13 shows that the statistical complexity with the Jensen–Shannon disbalance exhibits a satisfactory performance.

The results are summarized in Table 1. The checkmark indicates the possibility of confident indication of the useful signal, and x indicates the lack of this.

## 6. Conclusions

The article proposed a method for indicating the appearance of a useful signal in a heavily noisy mixture based on the statistical complexity. The analytical formulas used to determine the disequilibrium and statistical complexity were obtained using entropy variation. The effectiveness of the proposed approach for two types of acoustic signals in comparison with other information metrics was shown for different models of added noise. For white noise, the appearance of deterministic signal was shown to be reliably detected for a very small SNR (≈−15 dB) when the statistical complexity based on the spectral distribution variance was used as the criterion. However, for more complex noise models, the use of the statistical complexity with the Jensen–Shannon disequilibrium was shown to have better efficiency. Both the time and frequency domains were considered for the entropy calculation. The criteria for signal detection in a heavy noise mixture based on time distributions were shown to be less informative than those based on spectral distribution. The connection between the statistical complexity criterion and the Neyman–Pearson approach for hypothesis testing was also discussed. Future work will be devoted to research on the information criteria based on two- and multidimensional distributions, and acoustic signals with realistic background noise will be considered.

## Figures and Tables

**Table 1:** Comparison of information criteria for different noise models and SNRs.

Noise Type	SNR (dB)	Entropy $H(p^{s})$	Statistical Complexity $C_{SQ}$	Statistical Complexity $C_{JSD}$
White	−10	✓	✓	✓
	−17	×	✓	×
Pink	−5	×	✓	✓
	−10	×	×	✓
Brown	−5	×	✓	✓
	−10	×	×	✓

## Data Availability

The humpback whale song example was downloaded from https://cis.whoi.edu/science/B/whalesounds/bestOf.cfm?code=AC2A (accessed on 25 September 2022).

## Abbreviations

The following abbreviations are used in this manuscript:

FFT	Fast Fourier Transform
SNR	Signal-to-noise ratio

## Appendix A

**Proof of Lemma** **1.** The difference between entropies for the distributions $p_{i}$ and $q_{i}$ gives the entropy variation δH:

$$\begin{array}{c} \delta H=H\left(q+\delta q\right)-H\left(q\right)=-\sum_{i=1}^{N}\left(q_{i}+\delta q_{i}\right)\log_{2}\left(q_{i}+\delta q_{i}\right)+\sum_{i=1}^{N}q_{i}\log_{2}q_{i}=\\ -\sum_{i=1}^{N}\left(q_{i}+\delta q_{i}\right)\log_{2}\left(q_{i}\left(1+\frac{\delta q_{i}}{q_{i}}\right)\right)+\sum_{i=1}^{N}q_{i}\log_{2}q_{i}. \end{array}$$

The property of the logarithm of the product and the regrouping of the summands allows the chain of equations to continue as follows:

$$\begin{array}{c} \delta H=-\sum_{i=1}^{N}\left(q_{i}+\delta q_{i}\right)\left(\log_{2}q_{i}+\log_{2}\left(1+\frac{\delta q_{i}}{q_{i}}\right)\right)+\sum_{i}^{N}q_{i}\log_{2}q_{i}=-\sum_{i=1}^{N}q_{i}\log_{2}\left(1+\frac{\delta q_{i}}{q_{i}}\right)\\ -\sum_{i=1}^{N}\delta q_{i}\left(\log_{2}q_{i}+\log_{2}\left(1+\frac{\delta q_{i}}{q_{i}}\right)\right)=-\sum_{i=1}^{N}\delta q_{i}\log_{2}q_{i}-\sum_{i=1}^{N}\left(q_{i}+\delta q_{i}\right)\log_{2}\left(1+\frac{\delta q_{i}}{q_{i}}\right). \end{array}$$

The first sum is equal to the difference between cross-entropy and entropy. The next transformation is decomposed into an infinite logarithm series, and the resulting sum is divided into two parts:

$$\begin{array}{c} \delta H=H\left(p,q\right)-H\left(q\right)-\sum_{i=1}^{N}\sum_{n=1}^{\infty }\left(q_{i}+\delta q_{i}\right)\frac{{\left(-1\right)}^{n+1}}{n\ln2}{\left(\frac{\delta q_{i}}{q_{i}}\right)}^{n}=H\left(p,q\right)-H\left(q\right)\\ -\sum_{i=1}^{N}\sum_{n=1}^{\infty }\frac{{\left(-1\right)}^{n+1}\delta q_{i}^{n}}{n\ln2q_{i}^{n-1}}-\sum_{i=1}^{N}\sum_{n=1}^{\infty }\frac{{\left(-1\right)}^{n+1}\delta q_{i}^{n+1}}{n\ln2q_{i}^{n}}. \end{array}$$

One summand corresponding to n=1 is removed from the first sum, and summation continues with n=2.

$$\begin{array}{c} \delta H=H\left(p,q\right)-H\left(q\right)-\sum_{i=1}^{N}\frac{\delta q_{i}}{\ln2}-\sum_{i=1}^{N}\sum_{n=2}^{\infty }\frac{{\left(-1\right)}^{n+1}\delta q_{i}^{n}}{n\ln2q_{i}^{n-1}}-\sum_{i=1}^{N}\sum_{n=1}^{\infty }\frac{{\left(-1\right)}^{n+1}\delta q_{i}^{n+1}}{n\ln2q_{i}^{n}}. \end{array}$$

The resulting summand is zero. Shifting the summation index of the first summation results in δH in the following form:

$$\begin{array}{c} \delta H=H\left(p,q\right)-H\left(q\right)+\sum_{i=1}^{N}\sum_{n=1}^{\infty }\frac{{\left(-1\right)}^{n+1}\delta q_{i}^{n+1}}{\left(n+1\right)\ln2q_{i}^{n}}-\sum_{i=1}^{N}\sum_{n=1}^{\infty }\frac{{\left(-1\right)}^{n+1}\delta q_{i}^{n+1}}{n\ln2q_{i}^{n}}=H\left(p,q\right)-H\left(q\right)\\ +\sum_{i=1}^{N}\sum_{n=1}^{\infty }\frac{{\left(-1\right)}^{n+1}\delta q_{i}^{n+1}}{\ln2q_{i}^{n}}\left(\frac{1}{n+1}-\frac{1}{n}\right)=H\left(p,q\right)-H\left(q\right)-\sum_{i=1}^{N}\sum_{n=1}^{\infty }\frac{\delta q_{i}^{n+1}{\left(-1\right)}^{n+1}}{q_{i}^{n}n\left(n+1\right)\ln2}. \end{array}$$

Another shift of the summation index leads to the Equation (18), which ends the proof of the Lemma. □
